# Prevalence of Joint Complaints in Patients with Celiac Disease: A Systematic Review and Meta-Analysis

**DOI:** 10.3390/jcm14113740

**Published:** 2025-05-27

**Authors:** Dimitri Poddighe, Gulsamal Zhubanova, Dinara Galiyeva, Kamilla Mussina, Anders Forss

**Affiliations:** 1 College of Health Sciences, VinUniversity, Gia Lam District, Hanoi 10000, Vietnam; 2 School of Medicine, Nazarbayev University, 5/1 Kerey and Zhanibek Khans Street, Astana 010000, Kazakhstan; gulsamal.zhubanova@nu.edu.kz (G.Z.); d.galiyeva@nu.edu.kz (D.G.); kamilla.mussina@nu.edu.kz (K.M.); 3 Division of Clinical Epidemiology, Department of Medicine, Solna, Karolinska Institutet, Box 281, SE-171 77 Stockholm, Sweden; 4 Centre for Digestive Health, Gastroenterology Unit, Department of Gastroenterology, Dermatovenereology and Rheumatology, Karolinska University Hospital, SE-171 76 Stockholm, Sweden

**Keywords:** arthralgia, arthritis, joint pain, extra-intestinal manifestations, celiac disease, adults, children, prevalence

## Abstract

**Background**: Current evidence suggests that joint complaints can represent an extra-intestinal manifestation in patients with Celiac Disease (CD) without any rheumatic comorbidity. However, the prevalence of joint complaints in the context of both CD and rheumatic disease is not known. The aim of this study was to estimate the prevalence of joint complaints in patients with CD. **Methods**: We searched Medline, Embase, Cochrane, and Web of Science databases for studies reporting joint complaints in patients with CD between 1 January 1990 and 26 November 2024. Search results were screened by two independent reviewers. The pooled prevalence of joint complaints was estimated in meta-analysis using a random effects model. We conducted stratified analyses by region, age (adults vs. children), and study sample size. The Joanna Briggs Institute Critical Appraisal Tool was used to evaluate the quality of included studies, and publication bias was assessed using a funnel plot and Egger’s test. The study protocol was pre-registered in the PROSPERO database and the results were reported according to the PRISMA guidelines. **Results**: A total of 7414 publications were rendered in the search. Of these, 226 were reviewed in full text and 27 were included in the meta-analysis. Among 6901 patients with CD without any concomitant rheumatic diagnosis, 530 had joint complaints, yielding a weighted pooled prevalence of 10.7% (95%CI: 6.9–15.1). In meta-regression analysis, no association between the prevalence of joint complaints and patients’ clinical characteristics or demographics was found. **Conclusions**: This meta-analysis indicates that joint complaints in patients with CD without any specific rheumatic comorbidity are not uncommon. Increased awareness of joint complaints in CD is important to improve the diagnosis and clinical care of these patients.

## 1. Introduction

Celiac disease (CD) is an immune-mediated disorder triggered by the dietary intake of gluten in genetically predisposed individuals [1]. While the diagnostic hallmark of CD is distinctly represented by atrophy of the (duodenal) intestinal villi, in combination with a seropositive CD autoantibody profile, the clinical manifestations of the disease are highly variable [2]. The clinical presentation of CD is commonly described with gastrointestinal symptoms, including chronic diarrhea, bloating, abdominal pain, and secondary malnutrition. However, CD can also cause symptoms like anemia, dermatitis herpetiformis, osteoporosis, neuropathy, endocrine alterations, and musculoskeletal complaints. Some patients exhibit only extra-intestinal manifestations and such have come to the fore in the past decades, particularly in pediatric patients. While patients with CD, due to the risk of malnutrition, are often monitored for osteoporosis, the effects of the disease on other parts of the musculoskeletal system are often less cared for [3,4,5].

Although patients with CD could be affected by comorbid rheumatic diseases leading to musculoskeletal manifestations (e.g., juvenile idiopathic arthritis [JIA], rheumatoid arthritis [RA], and other rheumatic/autoimmune diseases) [6,7], current evidence suggests that joint complaints without any underlying specific rheumatic condition can be observed in patients with CD [8,9]. The mechanisms contributing to joint involvement in CD is largely unknown. It has been hypothesized that gluten exposure may contribute to the development of joint complaints by activating multiple pathological pathways, including the induction of oxidative stress, modulation of the innate and adaptive immune system, and exertion of pro-inflammatory effects [10].

To our knowledge, the prevalence of joint complaints has not systematically been investigated in CD patients without any rheumatic comorbidity. The aim of this study is to estimate the prevalence of joint complaints in this specific pathological setting, through a systematic review and meta-analysis of the existing literature.

## 2. Materials and Methods

This systematic review and meta-analysis were reported according to the Preferred Reporting Items for Systematic Reviews and Meta-Analyses (PRISMA) guidelines [11]. A study protocol was pre-registered in the PROSPERO database (Protocol ID: CRD42024509292).

### 2.1. Search Strategy

**Figure 1:**
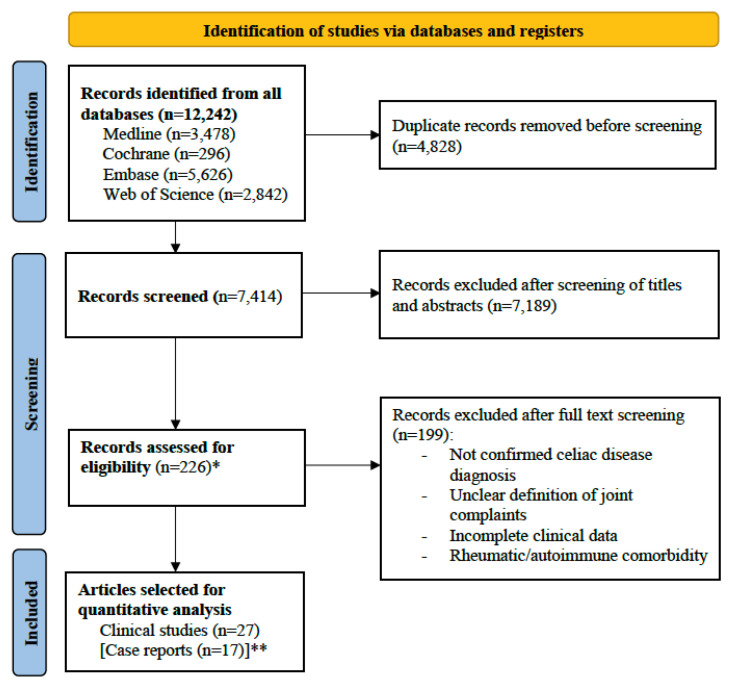
PRISMA flow diagram. * One additional publication was identified by screening of the reference list of the publications selected for quantitative analysis. Its full text was read but it was not deemed eligible for inclusion. ** Additional case reports were included as supporting clinical evidence in the interpretation of the meta-analysis findings.

We searched the databases of Medline, Embase, Cochrane, and Web of Science Core Collection from 1 January 1990 to 26 November 2024. The search strategy comprised the following MeSH terms: “pain”; “joint”; “celiac disease”; “glutens”; “transglutaminases”. Equivalent search strategies were applied for all databases (complete search strategies are presented in Table S1). Search strategies were elaborated in collaboration with librarians at the Karolinska Institutet University Library. We only included publications in the English language. Two reviewers (D.P and D.G.) independently screened all the search results. First, abstract and titles were screened. Second, the full text of eligible publications was screened (Figure 1). Finally, data were extracted from all the included studies according to a study-specific standardized extraction form. Reference lists of included studies were screened for additional publications not identified by our search strategies. Disagreement on inclusion and interpretation of studies or data was resolved by discussion between the reviewers, and in unresolved cases by mediation by the senior author. Additionally, to enrich our study with detailed clinical data not reported with sufficient granularity in the cohort studies included in the meta-analysis, we also screened the initial search results for case reports describing joint complaints in patients with CD. These reports can provide a clinical and diagnostic context to our findings, and allow better understanding of the potential association between joint complaints and CD.

### 2.2. Identification of Patients and Outcome

#### 2.2.1. Celiac Disease

For the diagnosis of CD, we required a reported small intestinal biopsy with Marsh stages II or III (or stage I with additional supporting evidence of CD). In studies where CD was reported as “biopsy-verified CD” or alike, the biopsy was presumed to be Marsh stage II or III.

#### 2.2.2. Joint Complaints

Joint complaints were defined as arthritis or arthralgia without a diagnosis of any defined rheumatic disease affecting the joints. Studies reporting joint complaints as joint pain or alike were also included if no concurrent diagnosis of any defined rheumatic disease affecting the joints was present.

### 2.3. Data Items and Risk of Bias

Data were retrieved on the following items: (i) first author and year of publication, (ii) country, (iii) study population size (i.e., number of CD cases in the study), (iv) proportion of females, (v) mean/median age and type of age group (children/adults), (vi) number of cases with joint complaints (including arthritis and arthralgia), and (vii) study design. The Joanna Briggs Institute (JBI) Critical Appraisal Tool was used to evaluate the quality of the included publications [12]. Potential publication bias was assessed using a funnel plot and Egger’s test.

As regards the case reports and small case series, the main individual demographic, clinical, and laboratory data for each reported case were extracted.

### 2.4. Statistical Analysis

We utilized the metaprop statistical command in STATA (StataCorp, College Station, TX, USA) to perform the meta-analyses. Metaprop, which is based on the “metan” command, pools effects, and employs specific measures using a binomial distribution to model variability within studies or the Freeman–Tukey double arcsine transformation to stabilize variances. This makes it suitable for analyzing proportions close to the extremes (0.0% and 100.0%). Heterogeneity between studies was assessed using Cochran’s Q-test and expressed as I^2^, with I^2^ > 50.0% indicating substantial heterogeneity. Leave-one-out analysis and an influence plot was used to further explore heterogeneity. Given the anticipated extensive heterogeneity among the included studies, we used a random effects model to estimate the weighted pooled prevalence. The significance level was set at *p* < 0.05.

We conducted subgroup analyses stratified by region (Asia: India and Uzbekistan; Europe: Finland, Italy, Kosovo, Netherlands, Spain, Sweden, Turkey, and United Kingdom; Middle East: Iran and Saudi Arabia; North and South America: Brazil and USA); adults vs. children; and study sample size (<100 vs. ≥100). We performed meta-regression analysis to explore the association between the prevalence of joint complaints and publication year, study sample size, proportion of females, and JBI Critical Appraisal tool score. These variables were selected based on their potential impact on the prevalence of joint complaints when comparing studies. All analyses were performed using STATA version 17 (StataCorp, College Station, TX, USA). Data extraction and compilation were conducted using Microsoft Excel (v.16.77.1, 2023, Microsoft Corporation, Redmond, WA USA).

### 2.5. Ethical Statement

This was a review study of the existing literature; hence, no ethical approval was required.

## 3. Results

### 3.1. Literature Research Output

Titles and abstracts of 7414 publications were screened (Figure 1). Of these, 226 were deemed relevant for full-text review. After full-text review, 199 publications were excluded and 27 fulfilled the eligibility criteria and were included in the main analysis (Figure S1). These 27 publications included a total of 6901 patients with CD with a female predominance (65% [four studies did not report the sex]). The characteristics of these publications are summarized in Table 1 [13,14,15,16,17,18,19,20,21,22,23,24,25,26,27,28,29,30,31,32,33,34,35,36,37,38,39].

### 3.2. Prevalence of Joint Complaints in Celiac Disease

Only studies reporting clinically or biopsy-verified CD (n = 27) were included in the main analysis. The mean study population for the included studies was n = 256 (standard deviation 307, range 8–1382), with 17 studies having a study population of ≥100 patients with CD. The majority of studies were retrospective studies (Table 1). All studies included clinical real-world data and the study periods spanned from 1980 [13] to 2022 [38]. Fourteen studies reported prevalence of joint complaints in children, ten studies in adults, and three studies in both adults and children.

**Figure 2:**
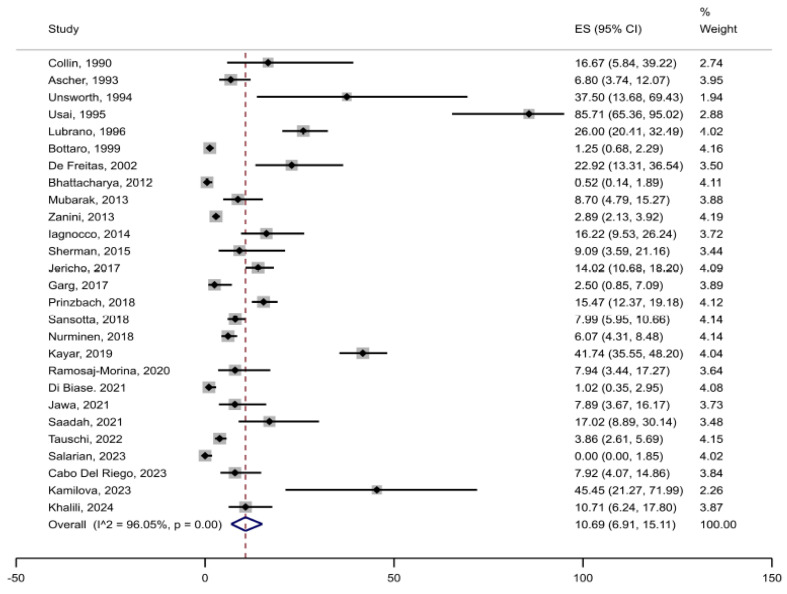
Prevalence of joint complaints in patients with confirmed celiac disease.

**Figure 3:**
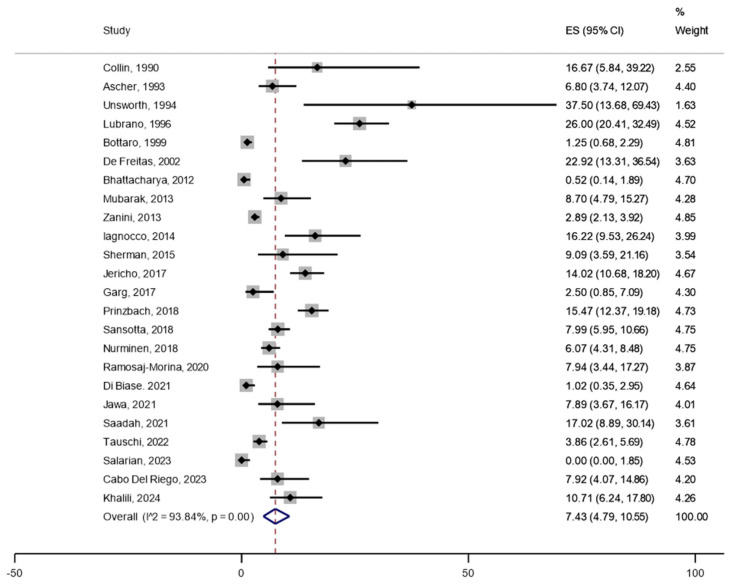
Prevalence of joint complaints in patients with confirmed celiac disease after excluding studies with a reported prevalence of >40%.

**Figure 4:**
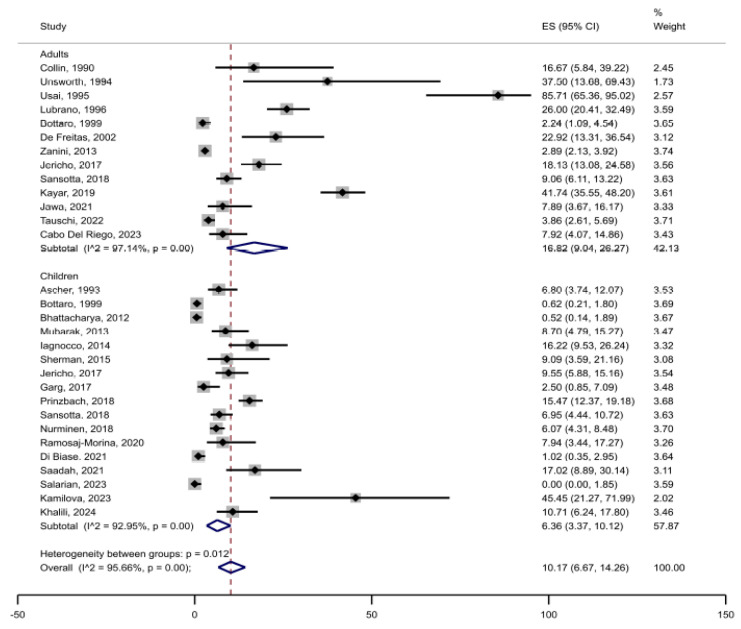
Prevalence of joint complaints in adults and children with confirmed celiac disease.

**Figure 5:**
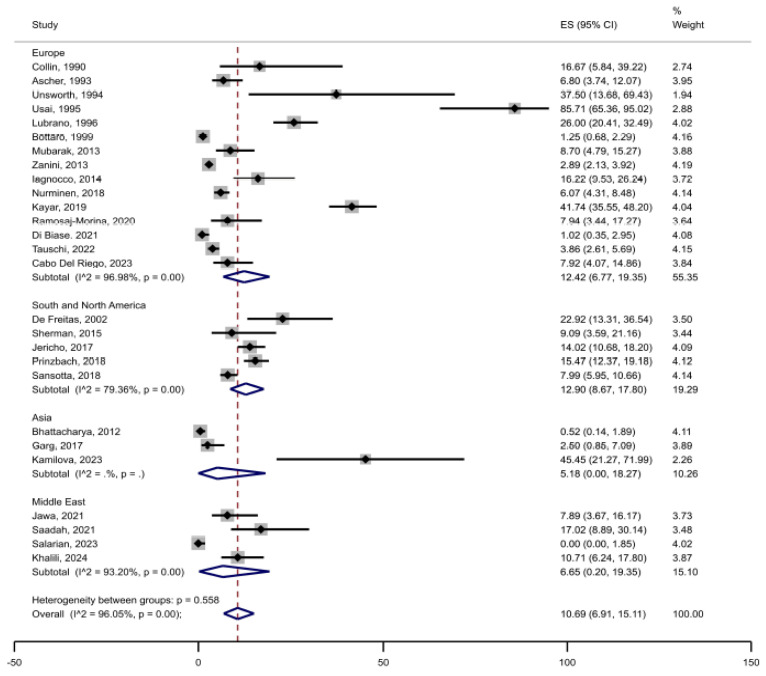
Prevalence of joint complaints in patients with confirmed celiac disease by region.

**Figure 6:**
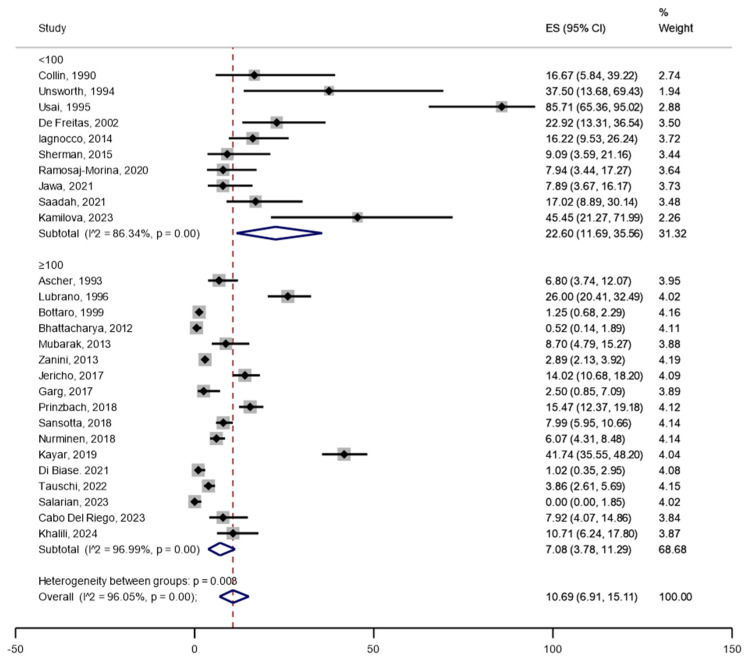
Pooled weighted prevalence of joint complaints in patients with confirmed celiac disease by study sample size <100 vs. ≥100.

Among a total of 6901 patients with CD included in the primary analysis, joint complaints (arthralgia or arthritis) were reported in 530 patients, rendering an unweighted crude prevalence of 7.7% in adults and children together (Table 1). The weighted pooled prevalence of joint complaints was 10.7% (95%CI 6.9 to 15.1) (Figure 2). The heterogeneity between studies was substantial (I^2^ = 96%, *p* < 0.05). Restricted to studies reporting prevalence of joint complaints of ≤40% (after excluding studies with remarkably high prevalence), the estimated weighted pooled prevalence was 7.4% (95%CI 4.8 to 10.6) (Figure 3). Stratified by adults (n = 17 studies) vs. children (n = 13 studies), the pooled prevalence was 16.8% (95%CI 9.0 to 26.3) in adults compared with 6.4% (95%CI 3.4 to 10.1) in children (Figure 4). In the comparison by region, studies in South and North America (n = 5) showed the highest weighted pooled estimate (12.9%; 95%CI 8.7 to 17.8); Europe (n = 15) had the second highest (12.4%; 95%CI 6.8 to 19.4, and Asia (n = 3) had the lowest prevalence (5.2%; 95%CI 0.0 to 18.3) (Figure 5). Stratified by study population size < 100 (n = 10) vs. ≥100 (n = 17), the weighted pooled prevalence was 22.6% (95%CI 11.7 to 35.6) compared with 7.1% (95%CI 3.8 to 11.3) (Figure 6).

### 3.3. Risk of Bias and Heterogeneity

**Figure 7:**
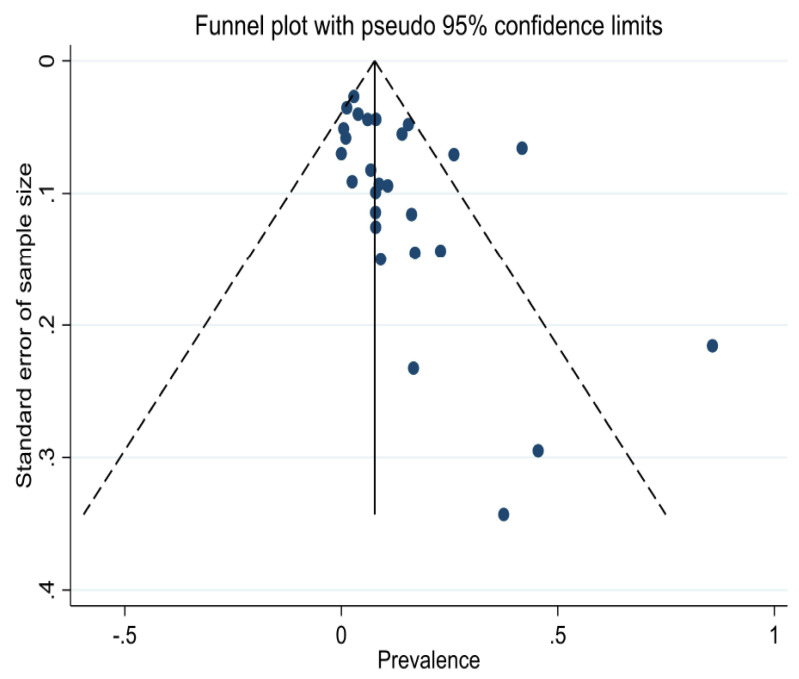
Funnel plot of included publications.

The funnel plot (Figure 7) indicated publication bias, with smaller studies showing higher prevalence (small-study effects). The Egger’s test was significant (*p* = 0.012), thus also indicating a small-study effect. The quality of included studies was assessed according to the JBI Critical Appraisal tool. The majority (n = 23/27) of studies showed a quality score of 8 or above (maximum score of 9), indicating generally high quality in the exposure and outcome measures as defined in this systematic review (Table S2). After excluding four studies [13,15,16,25] with a quality score lower than 8, the pooled weighted prevalence was 8.52% (95%CI 5.16 to 12.57) (Figure S1). To further investigate the high heterogeneity observed in the main analysis (I^2^ = 96%) and the influence of individual studies on the prevalence estimate, we conducted a leave-one-out analysis, recalculating the prevalence by excluding one study at a time. In this analysis, the pooled prevalence of joint complaints remained largely consistent, ranging from 9.14% to 11.49% (Table 2). We also explored potential sources of heterogeneity using an influence plot (Figure S2). Visual inspection identified three studies [18,22,30] as potential outliers. After excluding these studies, the pooled prevalence was 10.57% (95%CI 6.84 to 14.92) (Figure S3). Meta-regression analyses of the association between joint complaint prevalence in patients with CD and publication year (*p* = 0.069), study population size (*p* = 0.056), and proportion of females (*p* = 0.26) showed no significant associations (Figure S4A–C). However, meta-regression by JBI quality score showed an association (*p* < 0.001), with higher quality score (as a continuous variable) and lower prevalence of joint complaints (Figure S4D).

### 3.4. Case Reports

We identified a total of 17 case reports describing joint complaints in 18 patients with CD (Figure 1, Tables S3 and S4) [8,40,41,42,43,44,45,46,47,48,49,50,51,52,53,54,55]. Twelve cases involved females and five males, with sex not reported for one case. The age range was 7 to 80 years. Both mono-articular (n = 5) and poly-articular (n = 12) presentations were reported, with large joints more frequently affected than small joints. This pattern is consistent with ultrasound findings reported by Iagnocco et al. and Garg et al. [23,26]. Regarding laboratory parameters, there was substantial heterogeneity in the reported hematological and inflammatory markers. However, anemia was present in the majority of cases. Among inflammatory markers, the erythrocyte sedimentation rate was elevated in approximately half of the cases. These two findings may be related, as the erythrocyte sedimentation rate is influenced by hemoglobin levels [56]. In contrast, C-reactive protein levels and white blood cell counts were normal in nearly all cases. This may suggest that patients with CD and joint complaints do not exhibit systemic inflammation detectable in blood samples.

## 4. Discussion

Based on a total of 6901 patients from 27 studies, this systematic review and meta-analysis found a pooled weighted prevalence of joint complaints of 10.7% in patients with CD. To our knowledge, this is the first systematic review to assess the occurrence of joint complaints in patients with CD that are not related to other rheumatic or autoimmune comorbidities.

It is recognized that CD, through a variety of (immuno-)pathological mechanisms, can manifest with a wide range of extra-intestinal manifestations affecting the skin, liver, reproductive organs, and nervous system. Musculoskeletal manifestations are also seen in CD, and both arthritis and arthralgia are reported in patients with CD without any concomitant rheumatic disorder [2,9,57,58]. However, the knowledge about the prevalence and characteristics of such joint complaints as non-classical presentations of CD is limited. Moreover, joint manifestations in CD have primarily been investigated in the context of rheumatic diseases that were diagnosed as comorbidities in patients with CD, where JIA in children and RA in adults have been the main interest of most previous studies on this matter [59,60].

We found a 10.7% prevalence of joint complaints in patients with CD without any known rheumatic comorbidity. As a comparison, in a recent large population-based cohort study, Doyle et al. observed that both JIA and RA are 2- to 3-fold more common in patients with CD than in the general population [59]. The opposite relationship was investigated by Naddei et al., who reported a higher CD prevalence (2.4%) in a large cohort of patients with JIA compared to the general population [60]. A recent literature review suggested that at least 2.5% of patients with JIA are diagnosed with CD [7]. However, a recent meta-analysis of patients with JIA and RA did not find an increased risk of CD in these groups; notably, only biopsy-confirmed CD cases were included, which may have influenced the results for pediatric patients [6]. According to current European Society for Pediatric Gastroenterology, Hepatology, and Nutrition (ESPGHAN) guidelines, pediatric CD can also be diagnosed based solely on serologic markers under specific circumstances. Moreover, joint complaints can be caused by other rheumatic disorders, where a higher prevalence of CD (than that observed in the general population) has been reported, according to some meta-analyses and large cohort studies of patients with systemic lupus erythematosus [61], Sjogren Syndrome, and systemic sclerosis [62]. Conversely, there are several studies that found no increased prevalence of adult and pediatric CD in these rheumatic disorders [63,64,65].

In our meta-analysis, we found that 1 in 10 patients with CD suffers from joint complaints. This indicates that joint involvement is indeed not rare in these patients. We showed a lower prevalence of joint complaints in children than in adults, although studies that specifically aimed to study extra-intestinal manifestations in CD, including joint complaints, found a higher prevalence in children. For instance, Iagnocco et al. prospectively enrolled 74 children (mean age 7.6 years) with CD [23]. Of these, 38 were newly diagnosed and on a gluten-containing diet (GCD) and 36 were on a gluten-free diet (GFD) for at least 6 months. During the study period, 12 patients (16.2%) reported arthralgia: seven and five were on a GFD and GCD, respectively. Notably, all children underwent articular ultrasound assessment, regardless of whether they reported joint complaints. The proportion of patients with signs of synovitis on ultrasound examination in one or more joints (n =18/74, 24.3%) was higher than that of patients complaining of arthralgia (n = 12/74, 16.2%). Of note, both the number of patients exhibiting synovitis and the total number of affected joints with abnormal alterations on ultrasound examination were significantly higher in patients on a GCD than in those on a GFD (synovitis: 36.8% among the GCD vs. 11.1% among the GFD; total number of affected joints: 28.9% vs. 6.9%, respectively) [23]. Garg et al. used a similar study design and inclusion criteria to compare the presence of arthralgia and joint abnormalities on ultrasound examination in patients with CD (on a GCD or GFD) [26]. They found a lower proportion of children with arthralgia compared to the study by Iagnocco et al. [23], with all cases of arthralgia found in the GCD group (n = 3/60, 5%). However, in the GCD group, ultrasound joint abnormalities were observed in a higher number of patients with CD (n = 19/60, 31.7%), which indicates that most of them were asymptomatic despite the identified ultrasound abnormalities [26].

The findings of these studies could also indicate a role of GFD in alleviating joint complaints in patients with CD. The pathophysiology behind joint involvement in CD remains poorly understood. However, some authors have hypothesized that gluten may exhibit pro-inflammatory properties, induce oxidative stress, and impair the function of various components of the innate and adaptive immune system [10]. These mechanisms could contribute to the development of joint symptoms as well as other extra-intestinal manifestations. Therefore, a GFD may be beneficial for patients with CD by eliminating a potentially harmful effect of gluten, that may go beyond its established role in triggering CD-related immuno-pathophysiological abnormalities.

From the synthesis of the case reports, a pattern of both mono-articular and poly-articular presentation, with more frequent involvement of large joints than small joints, was seen. This aligns with the ultrasound examination findings reported by Iagnocco et al. and Garg et al. [23,26]. There was substantial heterogeneity of reported hematological and inflammatory parameters, although anemia was detected in the majority of the cases. Of the inflammatory parameters, the erythrocyte sedimentation rate (ESR) was increased in half of the cases. Anemia and a higher frequency of ESR could indeed be correlated, considering that ESR is affected by the level of hemoglobin in the blood [56]. On the other hand, both C-reactive protein levels and white blood cell counts were normal in almost all reported cases, which may suggest that patients with CD and joint complaints do not show signs of significant systemic inflammation in blood samples.

When interpreting our results, it is important to note that the meta-analysis included articles published over an extensive period of time (1990–2024), during which various guidelines for detecting CD have been used and the availability of endoscopic examinations varied. The awareness of joint complaints as non-classical manifestations of CD may also have substantially varied over time. These factors may have influenced our results. Of note, only a few studies in our meta-analysis had joint complaints (including joint pain, arthralgia, and arthritis) as the primary outcome [17,23,24,26]. The majority of studies instead had other primary outcomes but reported joint complaints as baseline characteristics or secondary outcomes. Moreover, several studies might not have detected all joint complaints of the study subjects, especially manifestations that could have been perceived as unspecific or mild by both patients and physicians. For this reason, the true prevalence of joint complaints could be higher than that reflected in our estimates.

To explore the robustness of our results, we performed several sensitivity and meta-regression analyses. First, we restricted our analysis to studies reporting prevalence of joint complaints of ≤40% (after excluding studies with remarkably high prevalence) and observed a weighted pooled prevalence of 7.4% (Figure S2). Second, we stratified publications by geographic region, where South and North America showed the highest weighted pooled estimate (12.9%) (Figure 5). Third, in meta-regression analyses, the prevalence of joint complaints was not associated with publication year, study population size, or proportion of females in the study population, but higher JBI quality assessment score was associated with lower prevalence of joint complaints, indicating a small-study effect on the prevalence estimate. Finally, we stratified publications by study population size (n) and found a prevalence of 22.6% in studies with n < 100 vs. 7.1% in studies with n ≥ 100.

The included publications showed signs of publication bias (Figure 7) related to smaller studies showing higher prevalence estimates (Figure S4A) and the heterogeneity between studies was large (I^2^ = 96%, Figure 2). To explore potential sources of the high heterogeneity, we conducted a leave-one-out analysis and an influence plot analysis. The prevalence estimates did not vary substantially across these analyses, and no individual studies were identified as clear contributors to the observed heterogeneity. However, it is well established that heterogeneity, as quantified by the I^2^ statistic, is often elevated in meta-analyses of proportions. Importantly, a high I^2^ value does not necessarily indicate meaningful or problematic heterogeneity in the context of prevalence studies [66].

This meta-analysis has several strengths. First, four major databases (Medline, Embase, Cochrane, and Web of Science) were used to identify relevant studies, ensuring a comprehensive literature screening. Second, the study focused on joint complaints in clinically confirmed cases of CD to increase internal validity. Third, we performed several meta-regression analyses to explore potential associations between study characteristics and joint complaint prevalence in CD. Finally, we contrasted our findings with case reports reporting detailed clinical characteristics of patients with CD and joint complaints.

We acknowledge several limitations of our study. First, we were unable to account for important potential confounding factors—including comorbidities (such as fibromyalgia and musculoskeletal disorders), age, genetic background, CD duration and severity, physical activity, and dietary habits—in the analysis of the pooled prevalence of joint complaints. Second, data on joint complaints were lacking from several regions, including East Asia, Oceania, and Africa. Third, we did not have access to individual-level ethnicity data, and therefore our findings should be interpreted with caution when generalizing across different ethnic populations. Fourth, our results may underestimate the true prevalence of joint complaints, as some participants with elevated serological markers did not undergo biopsy and were classified as not having joint complaints in the primary analysis. Finally, we were unable to assess the influence of medication use and adherence to a GFD on prevalence estimates due to limited data on these variables in the included studies.

## 5. Conclusions

In conclusion, this meta-analysis found that 1 in 10 patients with CD experience joint complaints that are not linked to any specific rheumatic disease. Joint complaints were more than twice as common in adults as in children. Despite some important limitations, such as concerns about the heterogeneity of included studies, our study suggests that joint complaints are extra-intestinal manifestations of CD that occur more frequently than previously thought. Therefore, patients with unexplained joint complaints, who do not have any rheumatic disease, may benefit from serological screening for CD, even if they do not exhibit gastrointestinal symptoms. Increased awareness of these joint complaints is important to improve the diagnosis and clinical care of patients with CD.

## Figures and Tables

**Table 1:** Summary of publications included in the meta-analysis of joint complaints in patients with celiac disease.

Authors, Year	Study Design	Country	Patients with CD (n)	Age Group	Females (%)	Mean Age (Years)	Arthalgia (n)	Arthritis (n)	Joint Complaints Total [n (%)]
Collin et al., 1990 [13]	Retrospective cohort study	Finland	18	Adults	78	41.6	2	1	3 (17)
Ascher et al., 1993 [14]	Retrospective cohort study	Finland/ Sweden	147	Children	62	5.00	10	0	10 (7)
Unsworth et al., 1994 [15]	Retrospective cohort study	United Kingdom	8	Adults	75	51.8	3	0	3 (38)
Usai et al., 1995 [16]	Retrospective cohort study	Italy	21	Adults	71	36.7	18	0	18 (86)
Lubrano et al., 1996 [17]	Retrospective cohort study	Italy	200	Adults	77	34.8	0	52	52 (26)
Bottaro et al., 1999 [18]	Retrospective cohort study	Italy	485	Children	-	7.7	3	0	3 (1)
“	“	“	313	Adults	-	24.4	7	0	7 (2)
De Freitas et al., 2002 [19]	Retrospective cohort study	Brazil	48	Adults	67	41.0	11	0	11 (23)
Bhattacharya et al, 2012 [20]	Retrospective cohort study	India	381	Children	42	6.2	0	2	2 (1)
Mubarak et al., 2013 [21]	Retrospective cohort study	The Netherlands	115	Children	72	6.5	10	0	10 (9)
Zanini et al., 2013 [22]	Retrospective cohort study	Italy	1382	Adults	-	-	40	0	40 (3)
Iagnocco et al., 2014 [23]	Retrospective cohort study	Italy	74	Children	70	7.6	12	0	12 (16)
Sherman et al., 2015 [24]	Retrospective cohort study	USA	44	Children	84	9.8	4	0	4 (9)
Jericho et al., 2017 [25]	Retrospective cohort study	USA	157	Children	-	8.9	11	4	15 (10)
“	“	“	171	Adults	-	40.6	15	16	31 (18)
Garg et al., 2017 [26]	Cross-sectional study	India	120	Children	48	6.7	3	0	3 (3)
Prinzbach et al., 2018 [27]	Retrospective cohort study	USA	433	Children	61	9.5	67	0	67 (15)
Sansotta et al., 2018 [28]	Retrospective cohort study	USA	259	Children	66	-	18	0	18 (7)
“	“	“	254	Adults	78	-	23	0	23 (9)
Nurminen et al., 2018 [29]	Retrospective cohort study	Finland	511	Children	65	-	31	0	31 (6)
Kayar et al., 2019 [30]	Retrospective cohort study	Turkey	230	Adults	75	33.4	96	0	96 (42)
Ramosaj-Morina et al., 2020 [31]	Retrospective cohort study	Kosovo	63	Children	63	5.5	5	0	5 (8)
Di Biase et al., 2021 [32]	Retrospective cohort study	Italy	295	Children	-	6.4	0	3	3 (1)
Jawa et al., 2021 [33]	Retrospective cohort study	Saudi Arabia	76	Adults	58	30.5	0	6	6 (8)
Saadah et al., 2021 [34]	Retrospective cohort study	Saudi Arabia	47	Children	47	8.7	8	0	8 (17)
Tauschi et al., 2022 [35]	Retrospective cohort study	Finland	621	Adults	74	-	24	0	24 (4)
Salarian et al., 2023 [36]	Cross-sectional study	Iran	204	Children	62	8.1	0	0	0 (0)
Cabo Del Riego et al., 2023 [37]	Retrospective cohort study	Spain	101	Adults	68	63.0	8	0	8 (8)
Kamilova et al., 2023 [38]	Retrospective cohort study	Uzbekistan	11	Children	55	5.7	5	0	5 (45)
Khalili et al., 2024 [39]	Cross-sectional study	Iran	112	Children	54	6.8	12	0	12 (11)

**Table 2:** Leave-one-out analysis of the pooled weighted prevalence estimate of joint complaints in patients with celiac disease.

Study	Prevalence (%, 95% Confidence Interval) *	I^2^ (%) *
Collin, 1990 [13]	10.57 (6.77–15.02)	96.18
Ascher, 1993 [14]	10.90 (6.97–15.52)	96.20
Unsworth, 1994 [15]	10.42 (6.70–14.80)	96.16
Usai, 1995 [16]	9.14 (5.75–13.15)	95.73
Lubrano, 1996 [17]	10.07 (6.44–14.34)	95.75
Bottaro, 1999 [18]	11.34 (7.33–16.03)	95.79
De Freitas, 2002 [19]	10.31 (6.55–14.73)	96.12
Bhattacharya, 2012 [20]	11.40 (7.44–16.02)	95.92
Mubarak, 2013 [21]	10.80 (6.89–15.38)	96.19
Zanini, 2013 [22]	11.30 (7.10–16.26)	95.95
Iagnocco, 2014 [23]	10.49 (6.68–14.98)	96.15
Sherman, 2015 [24]	10.76 (6.89–15.29)	96.19
Jericho, 2017 [25]	10.57 (6.71–15.12)	96.04
Garg, 2017 [26]	11.15 (7.19–15.78)	96.18
Prinzbach, 2018 [27]	10.50 (6.67–15.00)	95.91
Sansotta, 2018 [28]	10.91 (6.88–15.66)	96.18
Nurminen, 2018 [29]	11.02 (6.96–15.80)	96.20
Kayar, 2019 [30]	9.34 (6.17–13.02)	94.57
Ramosaj-Morina, 2020 [31]	10.81 (6.93–15.37)	96.19
Di Biase, 2021 [32]	11.34 (7.35–15.99)	96.05
Jawa, 2021 [33]	10.82 (6.93–15.39)	96.19
Saadah, 2021 [34]	10.48 (6.68–14.96)	96.16
Tauschi, 2022 [35]	11.17 (7.07–15.99)	96.16
Salarian, 2023 [36]	11.49 (7.55–16.08)	95.96
Cabo Del Riego, 2023 [37]	10.83 (6.93–15.41)	96.19
Kamilova, 2023 [38]	10.19 (6.50–14.54)	96.13
Khalili, 2024 [39]	10.71 (6.83–15.26)	96.18

* Pooled weighted prevalence estimate and I^2^ value after excluding the study listed in the corresponding row.

## Data Availability

The original contributions presented in the study are included in the article, further inquiries can be directed to the corresponding authors.

## Supplementary Materials

The following supporting information can be downloaded at: https://www.mdpi.com/article/10.3390/jcm14113740/s1, Figure S1. Pooled weighted prevalence of joint complaints in patients with confirmed celiac disease after exclusion of studies with Joanna Briggs Institute Critical Appraisal Tool score of less than 8; Figure S2. Influence plot of included studies; Figure S3. Pooled weighted prevalence of joint complaints in patients with confirmed celiac disease after exclusion of studies considered as outliers in the influence plot (Figure S2); Figure S4A–D. Meta-regression by study publication year (A), study population size (B), and proportion of females in the study (C), and (D) Joanna Briggs Institute Quality Assessment score; Table S1. Search strategies and search results; Table S2. Quality assessment of included studies according to the Joanna Briggs Institute (JBI) Critical Appraisal Tool; Table S3. Clinical characteristics of case reports on joint complaints in patients with celiac disease; Table S4. Laboratory parameters of case reports on joint complaints in patients with celiac disease.
