# Supplementary material for: Prevalence of Joint Complaints in Patients with Celiac Disease: A Systematic Review and Meta-Analysis
Source: J Clin Med. 2025 May 27;14(11):3740. doi: 10.3390/jcm14113740 (PMC12156437; doi:10.3390/jcm14113740)
Supplement: Supplementary file 1 [file jcm-14-03740-s001.zip › jcm-3585489-supplementary.pdf]

Supplementary Material

The authors have provided this supplementary material to give readers additional information about their work:

Prevalence of joint complaints in patients with celiac disease: a systematic review and meta-analysis

Dimitri Poddighe, Gulsamal Zhubanova, Dinara Galiyeva, Kamilla Mussina, Anders Forss

Table of Contents

Supplementary Figures **2**

Figure S1. Pooled weighted prevalence of joint complaints in patients with confirmed celiac disease after exclusion of studies with Joanna Briggs Institute Critical Appraisal Tool score of less than 8. 2

Figure S2. Influence plot of included studies. 3

Figure S3. Pooled weighted prevalence of joint complaints in patients with confirmed celiac disease after exclusion of studies (n=3) considered as outliers in the influence plot (Figure S2). 4

Supplementary Tables **7**

Table S1. Search strategies and search results 7

1. Medline 7
2. Embase 8
3. Cochrane Library 9
4. Web of Science Core Collection 10

Table S2. Quality assessment of included studies according to the Joanna Briggs Institute (JBI) Critical Appraisal Tool 11

Table S3. Clinical characteristics of case reports on joint complaints in patients with celiac disease 12

Table S4. Laboratory parameters of case reports on joint complaints in patients with celiac disease 14

References 16

Supplementary Figures

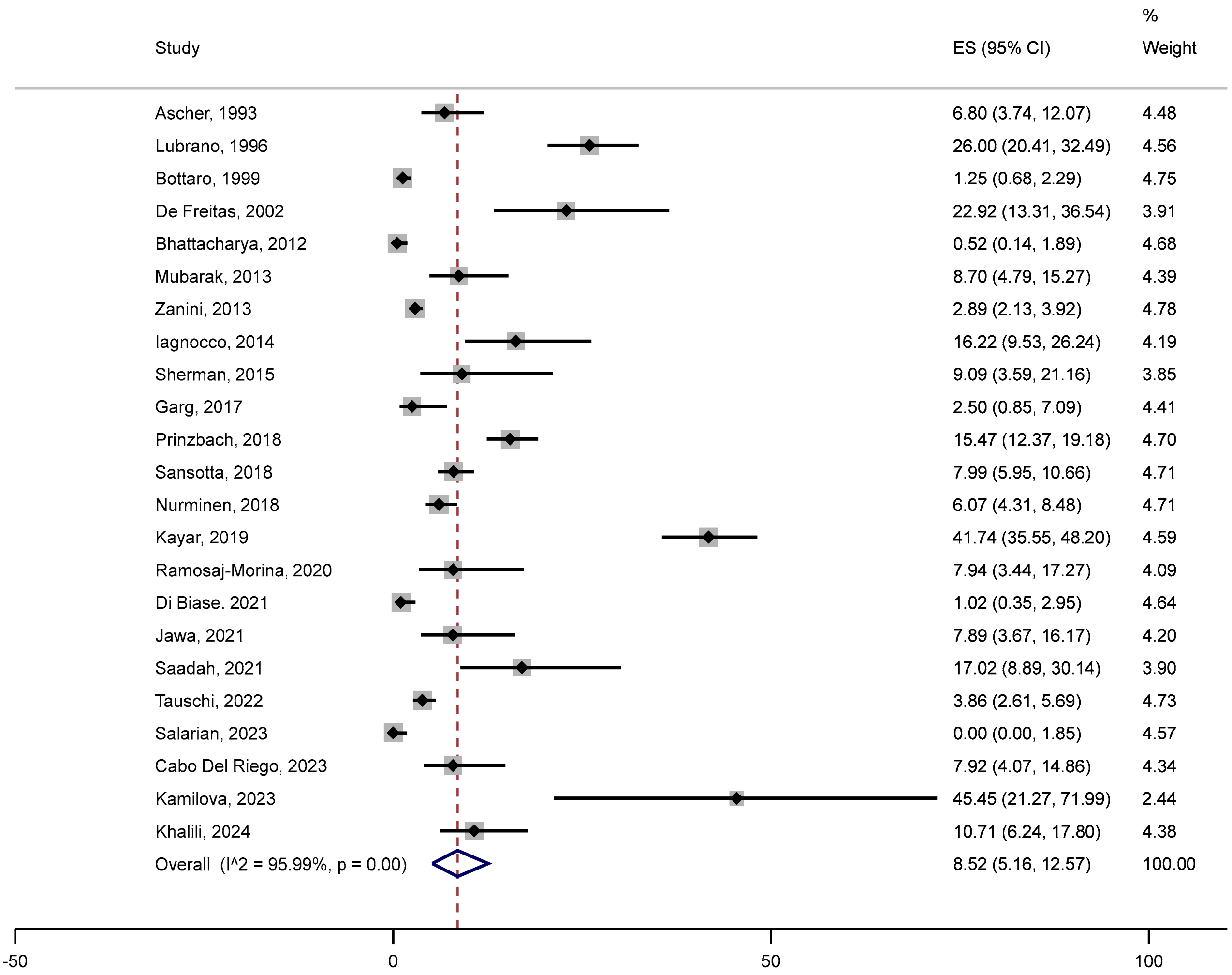

Figure S1. Pooled weighted prevalence of joint complaints in patients with confirmed celiac disease after exclusion of studies (n=4) with Joanna Briggs Institute Critical Appraisal Tool score of less than 8.

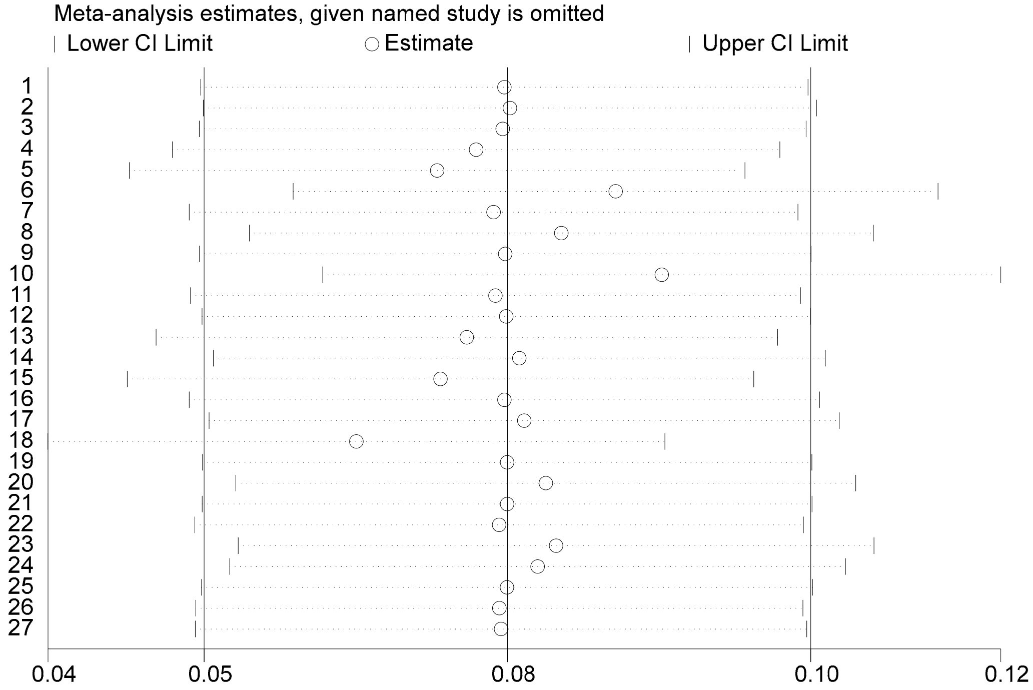

Figure S2. Influence plot of included studies.

*Studies 6 (Bottaro, et al), 10 (Zanini, et al), and 18 (Kayar, et al) were considered outliers by visual inspection.

.

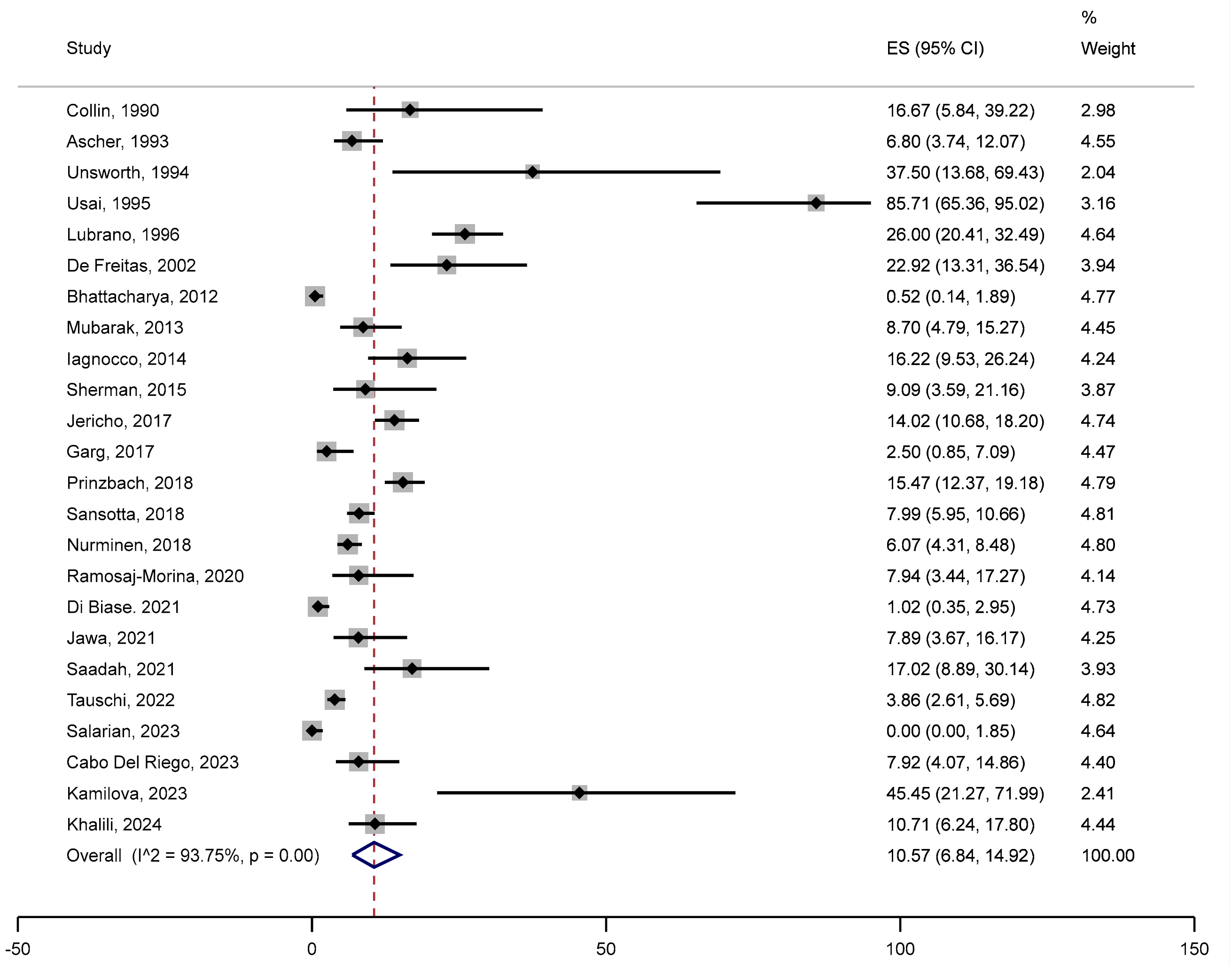

Figure S3. Pooled weighted prevalence of joint complaints in patients with confirmed celiac disease after exclusion of studies (n=3) considered as outliers in the influence plot (Figure S2).

A)

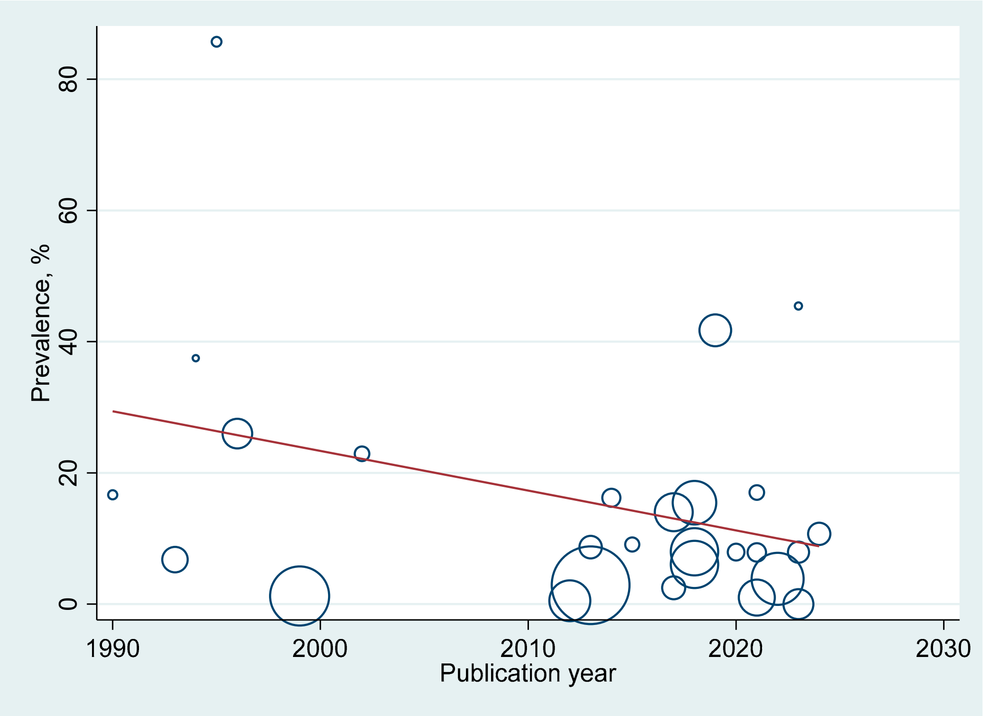

p=0.069; Regression coefficient (Publication year)= -0.61 (95%CI: -1.3 – 0.1); Beta-value= -0.36

B)

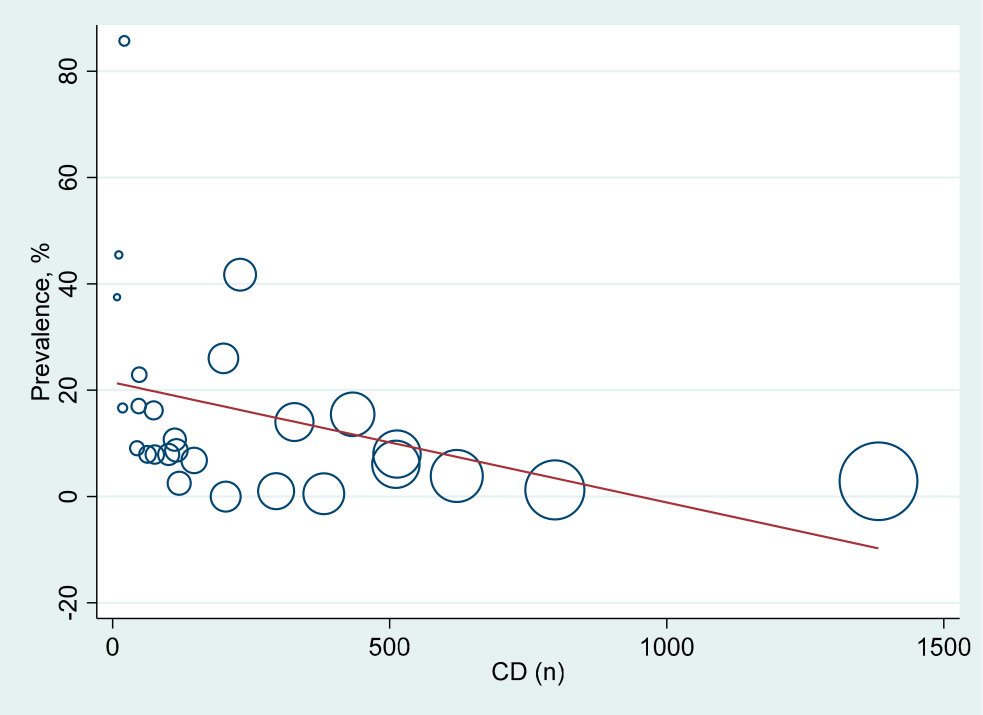

p=0.056; Regression coefficient (Celiac Disease, n)= -0.02 (95%CI: -0.05 – 0.001); Beta-value= -0.37

C)

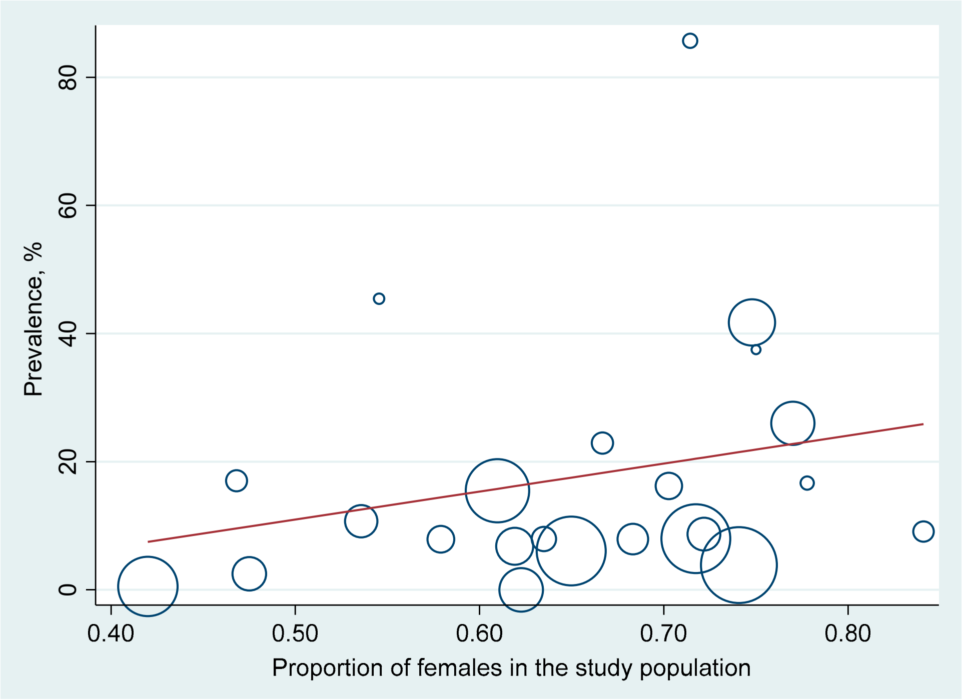

p=0.26; Regression coefficient (Proportion of females)= 43.7 (95%CI: -34.85 – 122.2); Beta-value= 0.24

D)

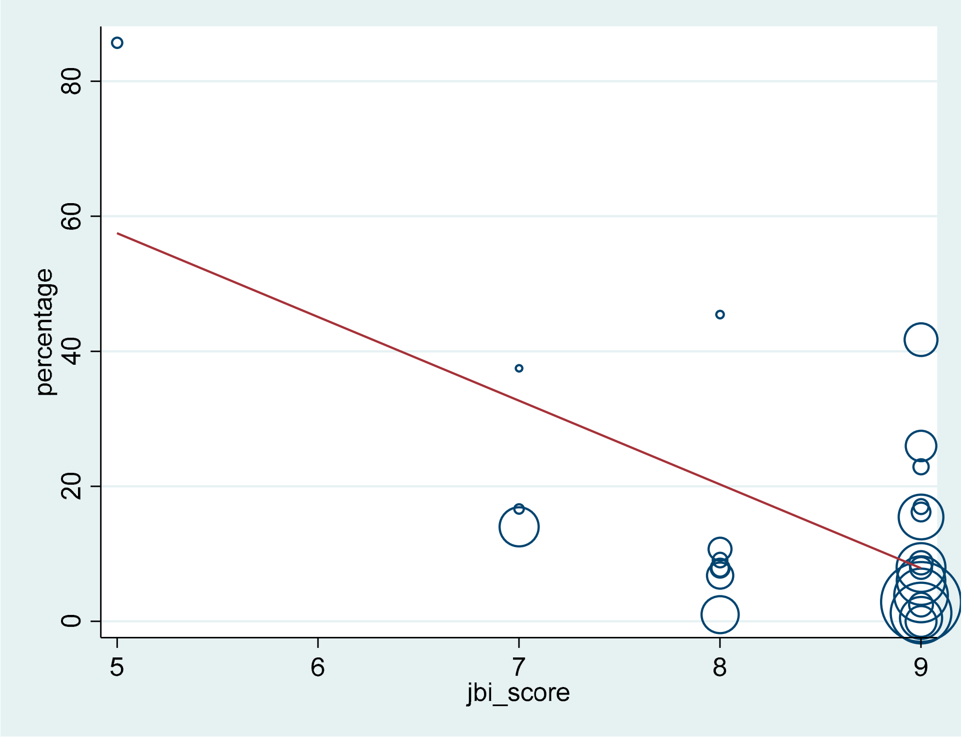

P < 0.001; Regression coefficient (JBI score)= -12.4 (95%CI: -18.5 – -6.3); Beta-value= -0.64

Figure S4 A-D. Meta-regression by: study publication year (A), study population size (B), and proportion of females in the study (C), and (D) Joanna Briggs Institute Quality Assessment Tool score.

Supplementary Tables

Table S1. Search strategies and search results

1. Medline

Interface: Ovid MEDLINE(R) ALL<br><br>Date of Search: Nov 26 2024<br><br>Number of hits: 3478<br><br>Comment: In Ovid, two or more words are automatically searched as phrases; i.e. no quotation marks are needed	Field labels<br><br>• exp/ = exploded MeSH term<br>• / = non exploded MeSH term<br>• .ti,ab,kf. = title, abstract and author keywords<br>• adjx = within x words, regardless of order<br>• * = truncation of word for alternate endings

Ovid MEDLINE(R) ALL <1946 to November 25, 2024>

1	exp Joint Diseases/	449325
2	exp Rheumatic Diseases/	272355
3	exp Joints/	290089
4	(art?ros* or diart?rosis or joint* or art?ropath* or rheumat* or art?riti* or oligoart?riti* or polyart?riti* or art?ralgia* or beauvais).ti,ab,kf.	750228
5	((extraintestinal or extra intestinal) adj3 (manifestation* or symptom*)).ti,ab,kf.	3613
6	(articular adj1 (affliction* or defect* or deformit* or disease* or disorder*)).ti,ab,kf.	1550
7	or/1-6	1024004
8	Celiac Disease/	22559
9	exp Glutens/	10184
10	exp Transglutaminases/	7973
11	(c?eliac* or c?eliak* or non-tropical sprue or nontropical sprue).ti,ab,kf.	33976
12	(aga or antigliadin* or gluten* or gliadin* or wheat).ti,ab,kf.	99515
13	(endomys* or antiendomys* or ema or aea or transglutamin* or anti-transglutamin* or trans glutamin* or ttg or tta or tgm2 or tgase).ti,ab,kf.	31924
14	((villus or villous) adj3 atroph*).ti,ab,kf.	2933
15	or/8-14	158108
16	7 and 15	3703
17	limit 16 to yr="1990-current"	3478

2. Embase

Interface: embase.com<br><br>Date of Search: Nov 26 2024<br><br>Number of hits: 5626<br><br>Comment: Emtree is the controlled vocabulary in Embase	Field labels<br><br>• /exp = exploded Emtree term<br>• /de = non exploded Emtree term<br>• ti,ab,kw = title, abstract and author keywords<br>• NEAR/x = within x words, regardless of order<br>• * = truncation of word for alternate endings

No.	Query	Results
#19	#17 NOT #18	5626
#18	#7 AND #15 AND [1990-2024]/py AND ([conference abstract]/lim OR [conference paper]/lim OR [conference review]/lim)	2613
#17	#7 AND #15 AND [1990-2024]/py	8239
#16	#7 AND #15	8459
#15	#8 OR #9 OR #10 OR #11 OR #12 OR #13 OR #14	199515
#14	((villus OR villous) NEAR/3 atroph*):ti,ab,kw	4540
#13	endomys*:ti,ab,kw OR antiendomys*:ti,ab,kw OR ema:ti,ab,kw OR aea:ti,ab,kw OR transglutamin*:ti,ab,kw OR 'anti transglutamin*':ti,ab,kw OR 'trans glutamin*':ti,ab,kw OR ttg:ti,ab,kw OR tta:ti,ab,kw OR tgm2:ti,ab,kw OR tgase:ti,ab,kw	47751
#12	aga:ti,ab,kw OR antigliadin*:ti,ab,kw OR gluten*:ti,ab,kw OR gliadin*:ti,ab,kw OR wheat:ti,ab,kw	112339
#11	c$eliac*:ti,ab,kw OR c$eliak*:ti,ab,kw OR 'non-tropical sprue':ti,ab,kw OR 'nontropical sprue':ti,ab,kw	51761
#10	'gliadin'/de	4801
#9	'gluten'/de	12410
#8	'celiac disease'/de	42228
#7	#1 OR #2 OR #3 OR #4 OR #5 OR #6	1629162
#6	(articular NEAR/1 (affliction* OR defect* OR deformit* OR disease* OR disorder*)):ti,ab,kw	2218
#5	((extraintestinal OR 'extra intestinal') NEAR/3 (manifestation* OR symptom*)):ti,ab,kw	6772
#4	art$ros*:ti,ab,kw OR diart$rosis:ti,ab,kw OR joint*:ti,ab,kw OR art$ropath*:ti,ab,kw OR rheumat*:ti,ab,kw OR art$riti*:ti,ab,kw OR oligoart$riti*:ti,ab,kw OR polyart$riti*:ti,ab,kw OR art$ralgia*:ti,ab,kw OR beauvais:ti,ab,kw	1033332
#3	'joint'/exp	259834
#2	'rheumatic disease'/exp	335235
#1	'arthropathy'/exp	1048264

3. Cochrane Library

Interface: Wiley<br><br>Date of Search: Nov 26 2024<br><br>Number of hits: 296	Field labels<br><br>• ti,ab,kw = title, abstract and author keywords<br>• NEAR/x = within x words, regardless of order<br>• * = truncation of word for alternate endings

ID	Search	Hits
#1	[mh "Joint Diseases"]	30053
#2	[mh "Rheumatic Diseases"]	22467
#3	[mh Joints]	11964
#4	(art?ros*:ti,ab,kw OR diart?rosis:ti,ab,kw OR joint*:ti,ab,kw OR art?ropath*:ti,ab,kw OR rheumat*:ti,ab,kw OR art?riti*:ti,ab,kw OR oligoart?riti*:ti,ab,kw OR polyart?riti*:ti,ab,kw OR art?ralgia*:ti,ab,kw OR beauvais:ti,ab,kw)	86996
#5	((extraintestinal:ti,ab,kw OR "extra intestinal":ti,ab,kw) NEAR/3 (manifestation*:ti,ab,kw OR symptom*:ti,ab,kw))	188
#6	(articular:ti,ab,kw NEAR/1 (affliction*:ti,ab,kw OR defect*:ti,ab,kw OR deformit*:ti,ab,kw OR disease*:ti,ab,kw OR disorder*:ti,ab,kw))	122
#7	#1 OR #2 OR #3 OR #4 OR #5 OR #6	97604
#8	[mh ^"Celiac Disease"]	502
#9	[mh Glutens]	237
#10	[mh Transglutaminases]	69
#11	(c?eliac*:ti,ab,kw OR c?eliak*:ti,ab,kw OR "non-tropical sprue":ti,ab,kw OR "nontropical sprue":ti,ab,kw)	1654
#12	(aga:ti,ab,kw OR antigliadin*:ti,ab,kw OR gluten*:ti,ab,kw OR gliadin*:ti,ab,kw OR wheat:ti,ab,kw)	4184
#13	(endomys*:ti,ab,kw OR antiendomys*:ti,ab,kw OR ema:ti,ab,kw OR aea:ti,ab,kw OR transglutamin*:ti,ab,kw OR anti-transglutamin*:ti,ab,kw OR ("trans" NEXT glutamin*):ti,ab,kw OR ttg:ti,ab,kw OR tta:ti,ab,kw OR tgm2:ti,ab,kw OR tgase:ti,ab,kw)	2173
#14	((villus:ti,ab,kw OR villous:ti,ab,kw) NEAR/3 atroph*:ti,ab,kw)	78
#15	#8 OR #9 OR #10 OR #11 OR #12 OR #13 OR #14	7090
#16	#7 AND #15 with Publication Year from 1990 to 2024, with Cochrane Library publication date Between Jan 1990 and Nov 2024, in Trials	296

4. Web of Science Core Collection

Interface: Clarivate Analytics<br><br>Editions = A&HCI , ESCI , SCI-EXPANDED , SSCI<br><br>Date of Search: Nov 26 2024<br><br>Number of hits: 2842	Field labels<br><br>• TS/Topic = title, abstract, author keywords and Keywords Plus<br>• NEAR/x = within x words, regardless of order<br>• * = truncation of word for alternate endings<br><br>Note: the *Exact search*-function was used for all the searches

#	Search Query	Results
1	TS=(art$ros* OR diart$rosis OR joint* OR art$ropath* OR rheumat* OR art$riti* OR oligoart$riti* OR polyart$riti* OR art$ralgia* OR beauvais)	1182946
2	TS=((extraintestinal OR "extra intestinal") NEAR/3 (manifestation* OR symptom*))	3881
3	TS=(articular NEAR/1 (affliction* OR defect* OR deformit* OR disease* OR disorder*))	4133
4	#1 OR #2 OR #3	1187723
5	TS=(c$eliac* OR c$eliak* OR "non-tropical sprue" OR "nontropical sprue")	41089
6	TS=(endomys* OR antiendomys* OR ema OR aea OR transglutamin* OR anti-transglutamin* OR "trans glutamin*" OR ttg OR tta OR tgm2 OR tgase)	45385
7	TS=((villus OR villous) NEAR/3 atroph*)	3082
8	#5 OR #6 OR #7	82532
9	#4 AND #8	2894
10	#4 AND #8 Timespan: 1990-01-01 to 2024-11-30	2842

Table S2. Quality assessment of included studies according to the Joanna Briggs Institute (JBI) Critical Appraisal Tool

**Author, publication year**	**Assessment (yes/no) by question[b]**									
	**Q1**	**Q2**	**Q3**	**Q4**	**Q5**	**Q6**	**Q7**	**Q8**	**Q9**	**Total number of yes**
Ascher et al, 1993	Y	N	Y	Y	Y	Y	Y	Y	Y	8
Bhattacharya et al, 2012	Y	Y	Y	Y	Y	Y	Y	Y	Y	9
Bottaro et al, 1999	Y	Y	Y	Y	Y	Y	Y	Y	Y	9
Cabo del Riego et al, 2023	Y	Y	Y	Y	Y	Y	Y	Y	Y	9
Collin et al, 1990	Y	N	N	Y	Y	Y	Y	Y	Y	7
De Freitas et al, 2002	Y	Y	Y	Y	Y	Y	Y	Y	Y	9
Di Biase et al, 2021	Y	Y	Y	Y	N	Y	Y	Y	Y	8
Garg et al, 2017	Y	Y	Y	Y	Y	Y	Y	Y	Y	9
Iagnocco et al, 2014	Y	Y	Y	Y	Y	Y	Y	Y	Y	9
Jawa et al, 2021	Y	Y	Y	Y	Y	Y	N	Y	Y	8
Jericho et al, 2017	Y	Y	Y	Y	N	Y	Y	Y	N	7
Kamilova et al, 2024	Y	Y	Y	Y	Y	Y	Y	Y	Y	8
Kayar et al, 2019	Y	Y	Y	Y	Y	Y	Y	Y	Y	9
Khalili et al, 2024	Y	Y	Y	Y	Y	Y	Y	Y	Y	8
Lubrano et al, 1996	y	y	y	y	y	y	y	y	y	9
Mubarak et al, 2013	Y	Y	Y	Y	Y	Y	Y	Y	Y	9
Nurminen et al, 2018	Y	Y	Y	Y	Y	Y	Y	Y	Y	9
Prinzbach et al, 2018	Y	Y	Y	Y	Y	Y	Y	Y	Y	9
Ramosaj-Morina et al, 2020	N	Y	Y	Y	Y	Y	Y	Y	Y	8
Saadah et al, 2021	Y	Y	Y	Y	Y	Y	Y	Y	Y	9
Salarian et al, 2023	Y	Y	Y	Y	Y	Y	Y	Y	Y	9
Sansotta et al, 2018	Y	Y	Y	Y	Y	Y	Y	Y	Y	9
Sherman et al, 2015	y	y	N	y	y	y	y	y	y	8
Tauschi et al, 2021	Y	Y	Y	Y	Y	Y	Y	Y	Y	9
Unsworth et al, 1994	Y	Y	N	Y	Y	U	Y	Y	Y	7
Usai et al, 1995	U	N	N	N	Y	Y	Y	Y	Y	5
Zanoni et al, 2013	Y	Y	Y	Y	Y	Y	Y	Y	Y	9

Y=Yes; N=No

[a] Munn Z, Moola S, Lisy K, Riitano D, Tufanaru C. Chapter 5: Systematic reviews of prevalence and incidence. In: Aromataris E, Munn Z (Editors). JBI Manual for Evidence Synthesis. JBI, 2020
[b] Questions:
Q1. Was the sample frame appropriate to address the target population?
Q2. Were study participants sampled in an appropriate way?
Q3. Was the sample size adequate?
Q4. Were the study subjects and the setting described in detail?
Q5. Was the data analysis conducted with sufficient coverage of the identified sample?
Q6. Were valid methods used for the identification of the condition?
Q7. Was the condition measured in a standard, reliable way for all participants?
Q8. Was there appropriate statistical analysis?
Q9. Was the response rate adequate, and if not, was the low response rate managed appropriately?

Table S3. Clinical characteristics of case reports on joint complaints in patients with celiac disease

**Authors (Publication year) [Country]**	**Age**	**Sex**	**Clinical manifestations (main aspects)**							**CD diagnosis**		
			**Arthralgia/ joint pain**	**Arthritis/ swollen joint**	**Affected joints**	**GI symptoms**	**Weight loss**	**Extraintestinal manifestations**	**Fever**	**Serology**	**Biopsy (histology)**	**HLA DQ2**
McDonagh et al.[1] (1992) [United Kingdom]	54y	n/a	Y	Y	Polyarticular (large/small joints, symmetric)	N	Y	Dermatitis herpetiformis, Anemia	N	AGA-IgA+ AGA-IgG+ EMA+	Y (SVA)	n/a
Young et al.[2] (1993) [USA]	64y	F	Y	Y	Wrist, ankle, MCP, PIP (asymmetric)	Diarrhea, abdominal pain	n/a	Headache	Y	n/a	Y (SVA)	n/a
Summers et al.[3] (1993) [United Kingdom]	51y	F	Y	Y	Knee	N	n/a	Anemia	n/a	EMA-IgG+ EMA IgA+ AGA+	Y (TVA)	n/a
Khattak et al.[4] (1994) [United Kingdom]	31y	F	Y	Y	Ankles; generalized arthralgia	Intermittent diarrhea	Y	Anemia	n/a	n/a	Y (TVA) *Resolved after GFD	n/a
Borg et al.[5] (1994) [United Kingdom]	42y	M	Y	Y	Ankle	N	n/a	N	n/a	-	Y (SVA) *Improved after GFD	n/a
Enzenauer et al.[6] (1998) [USA]	33y	F	Poly-arthralgia	N	Lower back, ankles, knees, shoulders, hands	Diarrhea	Y	N	n/a	EMA+, AGA-IgG+, AGA-IgA-	Y (VA)	n/a
Falcini et al.[7] (1999) [Italy]	11y	M	Y	Y	Knee	N	n/a	N	n/a	EMA+ AGA-IgG+, AGA-IgA-	Y (VA)	DQ2+
Bagnato et al.[8] (2000) [Italy]	37y	F	Y	N	Hands, wrists, knees	N	n/a	N	Y	EMA+, AGA-IgG-AGA-IgA+	Y (SVA)	DQ2+
Hepburn et al.[9] (2000) [United Kingdom]	80y	F	Y	Y	Ankles	Diarrhea	Y	Anemia	n/a	EMA- AGA IgA +	Y (TVA)	n/a
Slot et al.[10] (2000) [Denmark]	50y	M	Y	Y	Knee	N	Y	Unspecific dermatitis	Y	EMA+, AGA-IgA +	Y (VA)	n/a
	21y	F	Y	N	Sacro-iliac	Y	n/a		n/a	EMA+	Y (VA)	n/a

Dawidowicz et al.[11] (2008) [France]	44y	F	Polyarthralgia	N	Limbs		n/a	Non-pruritic skin rash	n/a	AGA-IgG- AGA IgA+ tTG+	Y (PVA)	n/a
Efe et al.[12] (2010) [Turkey]	34y	F	Y	Y	Knees, PIP, MTP	N	n/a	N	Y	AGA-IgG-, AGA-IgA+ tTG+ EMA+	Y (VA)	n/a
Ozyemisci-Taskiran et al. [13] (2010) [Turkey]	42y	F	Y	Y	Knee (left)	N	n/a	Dermatitis herpetiformis	n/a	AG- IgA+ tTG-IgA+	n/a	n/a
Priyadarshini et al.[14] (2022) [USA]	52y	F	Y	Y	Both knees and ankles	N	N	Anemia	N	EMA+ tTG+	Y (SVA)	n/a
Evangelatos et al.[15] (2023) [Greece]	66y	M	Y	N	Hips and knees	N	N	N	N	tTG-IgA+	Y (SVA)	n/a
A.Mougui et al.[16] (2023) [Morocco]	28y	F	Y	Y	PIP, MTP, wrists, knees, ankles	N	N	N	Y	tTG -IgA+	Y (SVA)	n/a
Küçükalİ B. et al.[17] (2024) [Turkey]	7y	M	Y	N	n/a	N	YES	Skin rash, generalized myalgia	N	AGA IgG tTG-IgA tTG-IgG	Y (VA)	n/a

AGA, anti-gliadin antibody; CD, celiac disease; EmA, anti-endomysium antibody; F, female; GI, gastrointestinal; M, male; MCP, metacarpophalangeal joint; MTP, metatarsophalangeal joint; n, number; N, no; n/a, not available; PIP, proximal interphalangeal joint; SVA, subtotal villous atrophy; TVA, total villous atrophy; tTG, anti-tissue transglutaminase antibody; VA, villous atrophy; y, years; Y, yes

Table S4. Laboratory parameters of case reports on joint complaints in patients with celiac disease

**Authors (Publication year) [Country]**	**CD (age; sex)**	**Laboratory parameters**																
		**WBC** (/mcl)	**Hb (g/dl)**	**MCV (fl)**	**PLT (/mm³)**	**ESR (mm/h)**	**CRP** (mg/l)	**Ferritin (ng/ml)**	**Folate (µg/l)**	**Vitamin B12 (ng/l)**	**Vitami D (ng/L)**	**Ca (mmol/l)**	**P (mmol/l)**	**AST (U/l)**	**ALT (U/l)**	**ALP (U/l)**	**TSH** (mIU/l)	**Ig (g/l)**
McDonagh et al.[1] (1992) [United Kingdom]	54y; F	n/a	11	85	n/a	7	n/a	<5	N	N	n/a	N	N	N	N	n/a	n/a	N
Young et al.[2] (1993) [USA]	64y; F	14900	11	n/a	330	20	n/a	n/a	n/a	n/a	n/a	7.9 (mg/dl)	2.6 (mg/dl)	11	n/a	138	n/a	n/a
Summers et al.[3] (1993) [United Kingdom]	51; F	n/a	10	72	n/a	40	N	5	N	N	n/a	n/a	n/a	n/a	n/a	359	n/a	H
Khattak et al.[4] (1994) [United Kingdom]	31y; M	n/a	L	L	n/a	17	n/a	n/a	L	L	n/a	1,8	n/a	n/a	n/a	551	n/a	N
Borg et al.[5] (1994) [United Kingdom]	42y; M	n/a	8	56	n/a	58	54	5	N	N	n/a	n/a	n/a	n/a	n/a	126	n/a	IgM =N IgG=N IgA=H
Enzenauer et al.[6] (1998) [USA]	33y; F	n/a	n/a	n/a	n/a	1	N	n/a	n/a	n/a	n/a	n/a	n/a	n/a	n/a	n/a	n/a	n/a
Falcini et al.[7] (1999) [Italy]	11y; M	n/a	N	N	N	N	N	n/a	N	n/a	n/a	N	N	N	N	n/a	n/a	n/a
Bagnato et al.[8] (2000) [Italy]	37y; F	11700	11	n/a	428	35	n/a	n/a	n/a	n/a	n/a	N	N	N	N	N	n/a	N
Hepburn et al.[9] (2000) [United Kingdom]	80y; F	10400	12*	92*	558	30	1	N*	92*	254*	n/a	1.5	1,2	N	N	323	n/a	N
Slot et al.[10] (2000) [Denmark]	50y; M	11300	N	122	n/a	n/a	28	n/a	L	n/a	n/a	N	N	N	N	N	N	N

	21y; F	2900	n/a	n/a	n/a	N	N	n/a	n/a	L	n/a	n/a	n/a	n/a	n/a	n/a	n/a	n/a
Dawidowicz et al.[11]<br>(2008)<br>[France]	44y; F	n/a	11	84	n/a	n/a	n/a	6	n/a	n/a	3.5	L*	N	n/a	n/a	n/a	6.73	n/a
Efe et al.[12]<br>(2010)<br>[Turkey]	34y; F	6700	11	69	278	125	47	5	n/a	n/a	n/a	N	N	N	N	N	n/a	n/a
Ozyemisci-Taskiran et al. [13]<br>(2010)<br>[Turkey]	42y; F	9200	11	n/a	n/a	75	11.7	n/a	n/a	n/a	5,1	N	N	N	N	N	N	n/a
Priyadarshini et al.[14]<br>(2022)<br>[USA]	52y; F	n/a	7	n/a	n/a	n/a	n/a	n/a	n/a	n/a	n/a	n/a	n/a	n/a	n/a	n/a	n/a	n/a
Evangelatos et al.[15]<br>(2023)<br>[Greece]	66y; M	n/a	11	82	n/a	34	6	7	n/a	210	5.2	2.7<br>(mg/dl)	8.5<br>(mg/dl)	n/a	n/a	335	n/a	n/a
~~A.~~Mougui et al.[16]<br>(2023)<br>[Morocco]	28y; F	8000	8	68	40	100	126<br>(mg/dl)	7	n/a	n/a	10.0	94<br>(mg/dl)	32<br>(mg/dl)	14	12	90	1,2	IgA=N
Küçükali ~~B.~~ et al.[17]<br>(2024)<br>[Turkey]	7y; M	6100	12	n/a	342	46	30.4	54 **	4.5	432	n/a	2.3	1.84	0.48<br>**	<0.15<br>**	n/a	n/a	n/a

AGA, anti-gliadin antibody; EmA, anti-endomysium antibody; F, female; n, number; H, high; L, low; N, normal; M, male; tTG, anti-tissue transglutaminase antibody.
* After supplementation
** Ferritin – 54 microg/L; AST – 0.48 ukat/L; ALT - <0.15 ukat/L

References

1. McDonagh JE, Griffiths ID. Arthritis--a presenting feature of occult coeliac disease. Br J Rheumatol. 1992; 31(12):857-8. doi: 10.1093/rheumatology/31.12.857-a.
2. Young TA, Hochman RF, Scopelliti JA. Celiac Disease and Arthropathy: Case Report and Literature Review. The Guthrie Journal 1993; 62(3), 99-104. Doi: 10.3138/guthrie.62.3.099
3. Summers GD, Hankey GL, Holmes GK. Oligoarthritis--a presenting feature of occult coeliac disease. Br J Rheumatol. 1993; 32(3):262. doi: 10.1093/rheumatology/32.3.262.
4. Khattak FH, Mattingly PC. Oligoarthritis associated with celiac-disease. Irish Med J 1994;.87(4), 115.
5. Borg AA, Dawes PT, Swan CH, Hothersall TE. Persistent monoarthritis and occult coeliac disease. Postgrad Med J. 1994; 70(819):51-3. doi: 10.1136/pgmj.70.819.51.
6. Enzenauer RJ, Root S. Arthropathy and celiac disease. J Clin Rheumatol. 1998;4(4):205-8. doi: 10.1097/00124743-199808000-00010.
7. Falcini F, Ferrari R, Simonini G, Calabri GB, Pazzaglia A, Lionetti P. Recurrent monoarthritis in an 11-year-old boy with occult coeliac disease. Successful and stable remission after gluten-free diet. Clin Exp Rheumatol. 1999; 17(4):509-11.
8. Bagnato GF, Quattrocchi E, Gulli S, Giacobbe O, Chirico G, Romano C, Purello D'Ambrosio F. Unusual polyarthritis as a unique clinical manifestation of coeliac disease. Rheumatol Int. 2000; 20(1):29-30. doi: 10.1007/s002960000061.
9. Hepburn AL, Kaye SA. Oligoarthritis in an elderly woman with diarrhoea and weight loss. Postgrad Med J. 2001; 77(909):475-7. doi: 10.1136/pmj.77.909.475.
10. Slot O, Locht H. Arthritis as presenting symptom in silent adult coeliac disease. Two cases and review of the literature. Scand J Rheumatol. 2000; 29(4):260-3. doi: 10.1080/030097400750041424.
11. Dawidowicz K, Ea HK, Lahalle S, Qubaja M, Lioté F. Unexplained polyarthralgia and celiac disease. Joint Bone Spine. 2008;75(3):325-8. doi: 10.1016/j.jbspin.2007.05.016
12. Efe C, Urün Y, Purnak T, Ozaslan E, Ozbalkan Z, Savaşs B. Silent celiac disease presenting with polyarthritis. J Clin Rheumatol. 2010;16(4):195-6. doi: 10.1097/RHU.0b013e3181dfcffd.
13. Ozyemisci-Taskiran O, Cengiz M, Atalay F. Celiac disease of the joint. Rheumatol Int. 2011;31(5):573-6. doi: 10.1007/s00296-010-1670-4
14. Priyadarshini S, Asghar A, Shabih S, Kasireddy V. Celiac Disease Masquerading as Arthralgia. Cureus. 2022; 14(6):e26387. doi: 10.7759/cureus.26387.
15. Evangelatos G, Kouna K, Iliopoulos A, Fragoulis GE. Musculoskeletal Complications of Celiac Disease: A Case-Based Review. Mediterr J Rheumatol. 2023 Mar 31;34(1):86-90. doi: 10.31138/mjr.34.1.86.
16. Mougui A, El Bouchti I. Isolated polyarthritis revealing celiac disease: A case report. SAGE Open Med Case Rep. 2023 Jul 8;11:2050313X231186305. doi: 10.1177/2050313X231186305.
17. Küçükalİ B, Bayrak H, Yıldırım DG, İnci A, Bakkaloğlu SA, Tümer L. A 7-year-old boy with scurvy owing to coeliac disease. Paediatr Int Child Health. 2024 Aug;44(2):63-67. doi: 10.1080/20469047.2024.2347001.
